# Interferon and Hepatitis B: Current and Future Perspectives

**DOI:** 10.3389/fimmu.2021.733364

**Published:** 2021-09-07

**Authors:** Jianyu Ye, Jieliang Chen

**Affiliations:** ^1^Key Laboratory of Medical Molecular Virology (MOE/NHC/CAMS), School of Basic Medical Sciences, Shanghai Medical College, Fudan University, Shanghai, China; ^2^Research Unit of Cure of Chronic Hepatitis B Virus Infection, Chinese Academy of Medical Sciences, Shanghai, China

**Keywords:** IFN, HBV, chronic hepatitis B, cccDNA, innate immunity, antiviral therapy, immunotherapy, biomarker

## Abstract

Chronic hepatitis B virus (HBV) infection remains a major health burden worldwide for which there is still no effective curative treatment. Interferon (IFN) consists of a group of cytokines with antiviral activity and immunoregulatory and antitumor effects, that play crucial roles in both innate and adaptive immune responses. IFN-α and its pegylated form have been used for over thirty years to treat chronic hepatitis B (CHB) with advantages of finite treatment duration and sustained virologic response, however, the efficacy is limited and side effects are common. Here, we summarize the status and unique advantages of IFN therapy against CHB, review the mechanisms of IFN-α action and factors affecting IFN response, and discuss the possible improvement of IFN-based therapy and the rationale of combinations with other antiviral agents in seeking an HBV cure.

## Introduction

Hepatitis B virus (HBV) leads to acute and chronic liver diseases that cause over 780000 deaths yearly worldwide and, currently, there are still more than 250 million chronically infected individuals (1). Chronic hepatitis B (CHB) can progress to cirrhosis in up to 40% of untreated patients, and there is an associated risk of decompensated cirrhosis and hepatocellular carcinoma (HCC) (2). There are two main antiviral therapies: nucleos(t)ide analogs (NAs) and pegylated interferon (IFN) α (PEG-IFN-α). NAs effectively control HBV replication but functional cure is rare. PEG-IFN has a limited treatment course and the responders to IFN therapy may maintain a virologic response after drug withdrawal, but its efficacy is still not satisfactory.

IFNs, a group of cytokines firstly described in 1957, are crucial modulators of the immune response against various viruses as well as carcinoma. IFNs are grouped into three types: I (α, β, ϵ, κ, ω), II (γ), and III (λ), based on the types of IFN receptors through which they signal. In humans, IFN-α can be further categorized into 13 different IFN-α subtypes, which all signal through a shared type I IFN heterodimeric receptor complex comprising two IFN-α receptor subunits (IFNAR1 and IFNAR2), and these IFNAR receptor subunits are present on nearly all nucleated cells (3). The IFN-IFNAR complex then activates the JAK-STAT pathway, resulting in the expression of dozens of interferon-simulating genes (ISGs), that function as downstream effectors to control viral replication and regulate immune responses. Here, we summarize the status of IFN-α-based therapy in CHB patients, review the mechanisms of IFN action and factors affecting responsiveness, and discuss the possible improvements in IFN therapy leading toward an HBV cure.

## Advantages and Mechanisms of IFN-α Treatment Against CHB

For CHB patients, standard PEG-IFN monotherapy is administered once weekly as a subcutaneous injection for 48 weeks, with advantages of finite treatment duration and sustained virologic response. PEG-IFN resulted in a sustained loss of hepatitis B e antigen (HBeAg) and nondetectable hepatitis in 30% of patients (4). In HBeAg-negative CHB, combination NAs plus PEG-IFN for 48 weeks is safe and could result in greater treatment efficacy than NAs monotherapy (5–7). For patients who achieve virologic suppression with NAs, such as entecavir (ETV), switching to a finite course of PEG-IFN significantly increases rates of HBsAg seroclearance to 20% of those with baseline HBsAg < 1500 IU/ml (8). In contrast, HBsAg seroclearance during NAs monotherapy is low (0-3% after 1 year) (9). Once the HBV genome was inactivated (“inactivate carriers”), HBsAg seroclearance occurred in 40% of those receiving PEG-IFN therapy (10, 11). In addition, treatment by PEG-IFN has been suggested to be associated with a lower incidence of HCC than NAs treatment in chronic HBV infection (12).

IFN-α treatment can induce an antiviral state in hepatocytes by regulating gene expression and protein translation, which exert non-cytolytic antiviral effects in several stages of the HBV life cycle.

First, HBV replicates its DNA genome through reverse transcription of its viral pregenomic RNA (pgRNA), and pgRNA is exported into the cytoplasm. In this process, the expression of APOBEC3 cytidine deaminases can be strongly enhanced by IFN-α simulation to induce extensive G-to-A hypermutations and block HBV DNA replication (13, 14). MX2, which is induced by IFN-α reduces HBV RNA levels by downregulating synthesis of viral RNA (15). IFN-α can also induce TRIM22, which binds to the HBV core promoter region to inhibit its transcription (16). In addition, IFN-α simulates ISG20, which binds to the HBV RNA terminal redundant region to degrade pgRNA (17, 18). IFN-α can also induce MyD88 to accelerate the degradation of pgRNA and promote the expression of MxA to impede HBV RNA nuclear export (19). Of note, HBV infection itself does not induce a significant ISG-mediated response in the liver. Liver biopsy samples from patients with HBV infection do not have higher levels of ISG expressions than those from patients without HBV infection (20). Moreover, recent *in vitro* studies suggested that HBV does not affect the pattern of ISG expressions induced by polyinosinic:polycytidylic acid (poly I:C) and Sendai virus (21). From this perspective, exogenous IFN-α may play unique roles in activating endogenous antiviral immune responses against HBV.

Second, once HBV delivers its 3.2kb rcDNA genome into the nuclei of hepatocyte, rcDNA can be repaired into the fully double-stranded covalently closed circular DNA (cccDNA) (22), which serves as the template for transcription of all viral mRNA. The HBV cccDNA is organized as a minichromosome in the nuclei of infected hepatocytes with various host and viral proteins, such as histone proteins and HBx (23, 24), and the intrahepatic cccDNA pool is not homogenous but exists as a heterogeneous population of viral minichromosomes (25). Accumulating evidence suggests that cccDNA transcriptional activity is regulated by epigenetic mechanisms (26, 27). HBx binds to the cccDNA and modifies the epigenetic landscape of cccDNA. The cccDNA without HBx expression transcribes significantly less pgRNA (28). Administration of IFN-α resulted in cccDNA-bound histone hypoacetylation as well as active recruitment to the cccDNA of transcriptional corepressors, which included reduction of acetylated histone H3 lysine 9 (H3K9) and 27 (H3K27) and increase in HDAC1 and Sirt1 in cccDNA minichromosomes. IFN-α treatment also reduced the binding of the STAT1 and STAT2 transcription factors to active cccDNA (29, 30). These modifications are associated with reduced transcription of pgRNA and subgenomic RNAs from the cccDNA minichromosome. In addition to regulating cccDNA transcription, recent studies suggested that IFN may induce degradation of cccDNA by inducing APOBEC3A and ISG20 (31, 32). However, it remains unclear how efficient such a mechanism is in the liver and whether cccDNAs in distinct epigenetic states are similar or differ in their sensitivity to IFN and IFN-induced antiviral factors. It is more certain that IFN-α affects cccDNA epigenetic modifications and represses cccDNA transcription, which in turn reduces the replenishment of the cccDNA pool. From this perspective, IFN-α can indirectly lead to subsequent reduction of the cccDNA pool (33). In addition, IFN-α treatment reduces the expression levels of HBx, which has been demonstrated to promote the degradation of the SMC5/6 complex to enhance HBV replication (34, 35), and thus can restore SMC5/6 expression, resulting in sustained cccDNA silence in HBV-infected human liver chimeric mice (36).

HBV-related protein translation and HBV virion secretion are two other processes that can be inhibited by certain ISGs. In the Huh-7 cell-based HBV transfection model (the double-stranded RNA (dsRNA)-dependent protein kinase) was induced by IFN-α treatment and then, reduced the replication-competent viral capsids, whereas the HBV transcripts, including pgRNA, were not affected (37). In addition, the IFN-inducible factor BST-2/tetherin was able to restrict HBV virion secretion. Knockdown of tetherin attenuated the IFN-α-mediated reduction of HBV virion release (38).

Beyond the scope of a single cell, cell-to-cell transmission of viral resistance is also a mechanism for amplifying IFN-α-induced antiviral response. IFN-α can induce the transfer of resistance to HBV from nonpermissive liver nonparenchymal cells (LNPCs) to hepatocytes *via* exosomes (39–41). Exosomes from IFN-α-treated LNPCs are rich in molecules with antiviral activity. Further studies suggest that macrophage exosomes depend on T cell immunoglobulin and mucin receptor 1 (TIM-1), a hepatitis A virus receptor, to enter hepatocytes for delivering IFN induced anti-HBV activity. Hepatocytes could utilize two primary virus infection endocytic routes (clathrin-mediated endocytosis (CME) and micropinocytosis) and lysobisphosphatidic acid (LBPA) to permit exosome entry and uncoating (42).

In addition to the direct anti-HBV effects, IFNs shape the landscape of the immune system to coordinate various immune cells. IFNs activated macrophages, natural killer cells, dendritic cells (DCs), and T cells. All these activated immune cells secrete a variety of cytokines, such as IL-1β, IL-6, TNF-α, and IFN-γ. Among them, IL-6, IL-12, and IL-15 by DCs were partially induced by IFN-α/β and then modulated B and T cell differentiation (Th1 polarization) and activation (43). IFN-I signaling in plasmacytoid dendritic cells (pDCs) led to altered CD69 and sphingosine-1-phosphate 4 (S1P4) receptor expression, which, in turn, affected pDCs retention in lymph nodes (44). IFN-α/β also enhance the antigen presenting capacity of the APC by increasing MHC class II, CD86, and CD40 expression. Moreover, IFN-α/β help neutrophil survival and strengthen phagocytosis of macrophage and neutrophil. HBV-specific CD8^+^ T cell are then propagated into the liver, and destructed the infected hepatocytes by perforin-granzymes or surface death receptors such as FAS/FASL, or both (45). This is called “cytopathic mechanism”.

Notably, HBV infection in immunocompetent adults is usually self-limited and transient. Over 90% of adults achieve viral control with strong, polyclonal, and multi-specific adaptive immune responses, such as specific CD8^+^ and CD4^+^ T cells, against HBV components (46). During acute HBV infection, most hepatocytes were reported to be infected by HBV. Assuming most infected hepatocytes are destroyed through the cytopathic mechanism, patients will have severe liver trauma, which is rarely seen in the clinic. Thus, “non-cytopathic mechanism” has been proposed to explain this (47). It is believed that non-cytopathic mechanisms allow infected hepatocytes to purge HBV replicative intermediates from the cytoplasm and cccDNA from the nucleus without being killed. Some clues support this notion. First, HBsAg-specific class I-restricted cytotoxic T lymphocytes (CTLs) profoundly suppress hepatocellular HBV gene expression in HBV transgenic mice by a noncytolytic process. This regulatory effect of the CTLs is initially mediated by IFN-α/β, IFN-γ, and TNF-α, which greatly exceeds their cytopathic effects in magnitude and duration (47, 48). Second, in acutely infected chimpanzees, HBV DNA was shown to largely disappear from the liver and the blood long before the peak of T cell infiltration (49). When knocking out one of these key components (T cell, NK cell, Fas, IFN-γ, IFN-α/β, and TNF-α) in the mouse model, hydrodynamically injected HBV-expressing plasmid persisted for at least 60 days, indicating that each of these effectors contributes to eliminate HBV components (50). In addition, proinflammatory cytokines such as IL-6, IL-1β, IL-4, and TGF-β, which could be induced by IFN show antiviral effects in different stages of HBV replication (51). Nonetheless, the non-cytopathic mechanism has not been adequately revealed because of the complexity of the liver immune microenvironment and the lack of an appropriate animal model.

In IFN-mediated control and clearance of HBV infection, it is still uncertain to what extent the direct anti-HBV effects and indirect immunomodulatory effects contribute to the IFN-α-mediated antiviral action. In the HBV-infected humanized uPA/SCID mice model, PEG-IFN-α treatment can induce sustained responsiveness in HBV-infected hepatocytes and trigger substantial HBV antigen decline without the involvement of immune cell response (52). Notably, a poor restoration of immune cell functions was observed in the early phases of IFN treatment (53), but changes in the inflammatory environment in the liver take a long time to develop and cytotoxic CD8+ T cells can be more readily expanded in the blood of treatment responders than nonresponders, indicating the importance of immune cells in HBV control and supporting a role of IFN-based therapy in restoration of the immune system.

One study indicated that the absolute number of CD8^+^ T cells were strikingly reduced, including CMV-specific CD8 T cells, while CD56^bright^ NK cells were potently expanded in a cohort of HBeAg negative patients receiving PEG-IFN-α therapy. Depleting CD8^+^ T cells may limit the efficacy of PEG-IFN-α, on the other hand, CD56^bright^ NK cells could enhance anti-HBV efficacy (54). More understanding of the mechanisms of IFN-α action will assist in the improvement of antiviral efficacy.

## Factors That Influence IFN Response

HBV has been called a ‘stealth’ virus since it does not induce a significant IFN response in the liver. Besides, sustained off-treatment response to exogenous IFN-α therapy can be achieved only in a minority of CHB patients. Both host and viral factors influence the IFN response during HBV infection and IFN-α therapy **(** **Table 1** **)**, some of which could be used as predictors to improve the cost-effectiveness of IFN-α therapy.

**Table 1:** Viral and host factors that affect IFN response during HBV infection and IFN therapy.

Viral and host factors			Role in modulating IFN response	Ref
Viral Factors	Viral load		Lower viral load and antigen levels associate with higher responsiveness to IFN-α therapy	(55, 56)
	Genotype and mutants		Gt A and B associates with better response to IFN treatment than gt D and C, respectively	(57–60)
			Precore and core promoter mutants limits the probability of response to IFN in HBeAg-positive CHB	
	Viral Counteractions	HBsAg	Inhibits with TLRs signaling and IFN-α induction in pDCs, and interfers with immune cell functions.	(61–63)
		Pol	Inhibits RIG-I/TLR3/STING-IRF-IFN-I and IFN-I-JAK/STAT signaling in hepatocytes	(64–66)
		HBx	Inhibits RIG-I/MAVS signaling	(67–69)
		HBc	Inhibits IFN-inducible MxA and IFITM1	(70, 71)
		HBeAg	Inhibits TLR2 and TIR intracellular form and IFN signaling	(72–74)
		HBSP	Inhibits IFN-I signaling	(75)
Host Factors	ALT		Higher baseline ALT levels are predictive of better IFN responsiveness	(56, 76)
	Age and gender		Younger age and female are predictive of better IFN responsiveness	(77, 78)
	Genetic polymorphisms		SNPs of IL28B(IFN-λ3), STAT4 and UBE2L3 could be associated with IFN response	(79–87)
	Liver stage and intrinsic sensitivity to IFN		Staging of liver fibrosis, presence of liver steatosis may affect the IFN response	(55, 78)
	Anti-IFN antibodies		May associate with non-response to IFN-α therapy	(88–91)

HBSP, hepatitis B spliced protein; IFITM1, interferon induced transmembrane protein 1; MxA, myxovirus resistance protein; RIG-I, retinoic acid-inducible gene I; TIR, Toll/IL-1 receptor; STING, stimulator of interferon genes; IRF, interferon regulatory factor; MAVS, mitochondrial antiviral-signaling protein.

## Viral Factors

Low HBV viral and antigen load is important predictor favoring IFN therapy. The viral load could influence both innate and adaptive immune response pathways, which impairs the response to IFN. In untreated CHB patients, intrahepatic gene expression profiling showed a strong downregulation of the antiviral effector, interferon stimulated genes, and pathogen recognition receptor pathway compared with non-infected controls (92). Although it remains controversial whether the host could detect the virion and express innate and IFN genes during HBV infection (20, 21, 93–97), HBV has developed strategies to counteract the innate and IFN system. HBV particles readily inhibit host innate immune responses upon virion/cell interaction (98, 99). HBV polymerase (Pol) and HBx could block multiple critical innate immune response pathways in hepatocytes, including RIG-I, STING, TRIM22, and IRF (64, 65, 100). HBsAg could inhibit the induction of IFN-α and proinflammatory cytokines such as IL-12, and HBeAg could target and suppress activation of the TLR and related signaling pathway (61, 62, 72). In addition, prolonged exposure of T cells to high quantities of HBV-related antigen, especially HBsAg, is the most likely reason for defective T-cell function in CHB patients (101). The decline of viral load is beneficial for the antigen removal, which allows T cells to rest from antigenic stimulation and might be necessary for reconstitution of functional T-cell responses (102, 103). Moreover, higher HBV replication levels lead to a lower sensitivity of HBV to IFN-α in PHH culture models (55), corresponding to the clinical observation that higher viral load is associated with poor response to IFN-α. In this regard, IFN-based therapy may contribute to the restoration of the immune system by directly suppressing antigen production in infected hepatocytes, in addition to its immunomodulatory effect. On the other hand, the magnitude of HBV-specific CD8+ T cell response is primarily regulated by the initial antigen expression level, and a recent study using the HBV hydrodynamic transduction model suggests that IFN-I signaling may negatively regulated HBV-specific CD8+ T cell responses by reducing early HBV antigen expression (104). Notably, although HBV *per se* does not activate IFN-I signaling during natural HBV infection, recent studies in chimpanzees and humanized mouse models indicate that IFN-I signaling induced by HCV infection could contribute to HBV control (105, 106).

HBV genotype has been shown to influence the therapeutic response to IFN-α therapy. In HBeAg-positive patients, the SVR was significantly better in genotypes A and B patients than for genotypes C and D that were treated with standard IFN-α (7). In HBeAg-positive Asian populations, HBV genotype B patients are more responsive to IFN-based therapy, whether PEG-IFN-α or standard IFN-α therapy, whereas genotype C patients have a higher likelihood of response to PEG-IFN-α compared to standard IFN-α (107). In HBeAg-negative patients, PEG-IFN-α was less effective in genotypes D and E with an SVR of around 20%. It should be noted that the prevalence of HBV genotypes varies geographically. Although genotypes A to J have been found (108), genotypes A, B, and C are most prevalent in North America (109). Genotypes B and C are the dominant types in East Asia (110). The above clinical statistical conclusions may be influenced by other factors, such as race, lifestyle, and, even, infected period. For instance, genotype A2 was mostly adult acquired whereas genotypes B and C were transmitted at birth or in very early childhood in a large proportion of Asian patients. The differential IFN response observed among these patients might be in part a reflection of transmission-related immune tolerance or host genetic polymorphisms, rather than the viral genotypes. Nonetheless, HBV genotyping is useful in patients being considered for IFN-α therapy (111). Since HBV population in the host usually consists of remarkable genetic heterogeneity and persists in the form of quasispecies, a number of studies have indicated that HBV mutations at baseline might affect IFN response (112, 113).

## Host Factors

A series of host factors have been identified as independent predictors of response to IFN therapy including younger age, female gender in HBeAg-negative CHB patients, and high serum alanine transaminase (ALT) levels (≥2-5 upper limit of normal) in both HBeAg-negative and positive patients (56, 76). Although high serum ALT levels may indicate active immune status and eradication of HBV, the mechanism behind its association with response to IFN is worth exploring.

Host genetics is an important aspect affecting the responsiveness to treatments. Interleukin (IL)28B polymorphisms were reported to be associated with IFN-α treatment response in CHB patients. The relationship between IL28B polymorphisms and response to PEG-IFN-α therapy has also been investigated. Among HBeAg-positive patients, three different single nucleotide polymorphisms (SNP) in IL28B were investigated separately: rs8099917, rs12980275, and rs8099917 (94–96). The polymorphisms in rs8099917 showed no difference in response to PEG-IFN-α, while rs12980275 and rs8099917 polymorphisms showed a difference in HBeAg seroconversion rate. Among HBeAg-negative patients, one study enrolled 101 patients treated with PEG-IFN-α therapy for a median of 23 months (79). Most patients were middle-aged men with HBV genotype D, average serum HBV DNA 6.0 log cp/mL, ALT 136 IU/L, and 42% with cirrhosis. The proportion of patients with CC, CT, and TT genotypes for IL28B rs12979860 were 47%, 42%, and 11%, respectively. The rate of serum HBsAg clearance was 29% in CC compared to 13% in non-CC. The C allele of rs12979860 was associated with a higher rate of response in HBeAg-negative patients with genotype D of HBV treated with PEG-IFN-α. However, the power of IL28B polymorphisms to predict the outcomes of IFN therapy remains limited because of various SNPs, patients’ conditions, and duration of treatment.

Given that STAT4 is an important part of the JAK-STAT pathway, SNPs in STAT4 could also be candidates that predict the outcome of IFN-α therapy. Multiple genomic loci, rs7574865, rs4274624, rs11889341, rs10168266, and rs8179673, were shown to be associated with the risk of HBV infections or predictors of PEG-IFN-α therapy (80). For instance, in HBeAg-positive patients, the rs7574865 GG genotype was significantly associated with a reduced sustained virologic response (SVR, defined as HBeAg seroconversion and HBV DNA level < 1000 cp/ml) compared with the GT/TT genotype in patients receiving PEG-IFN-α (18.0% *versus* 41.2%, P = 9.74×10^-5^) or IFN-α (21.1% *versus* 37.2%, P = 0.01) therapy (81). However, meta-analysis cannot validate the correlation between STAT4 rs7574865 and HBV susceptibility or natural clearance (82, 83). To sum up, transferring these genomic approaches to clinical practice to improve pretreatment patient selection is still challenging.

The occurrence of endogenous anti-IFN antibodies is another factor that may be associated with non-response to IFN-α therapy. During IFN therapy, IFN neutralizing antibodies appeared in the serum of 7%~39% CHB patients (88–90). Anti-IFN antibodies lowered the levels of serum IFN bioactivity and may reduce downstream IFN signaling pathways. For patients relapsing during or after IFN treatment, the appearance of anti-IFN antibodies is likely to occur prior to or at the same time as serum HBV DNA loss (88). However, a study retrospectively suggested that the presence of anti-IFN antibodies was not associated with non-response to PEG-IFN-α therapy in CHB patients (91). In this regard, more studies are required to identify the role of anti-IFN antibodies in the process of HBV chronic infection. In addition, since IFN-α can be divided into different subtypes, it would be interesting to know whether the anti-IFN-α antibodies have subtype bias.

Several cell culture systems have been used to study HBV and IFN interaction. For liver cell culture models, there are mainly four types of systems: hepatoma cell lines (such as HepG2 and Huh7) and related strains (such as HepG2-NTCP and HepAD38), bi-potent liver progenitor cell line (HepaRG), primary human hepatocytes (PHH) (114), and induced human hepatocyte-like cells (such as HepLPCs and iHeps) (115–117). Notably, the responses of IFN differ a lot among these models. Among them, PHH is still regarded as the gold standard for hepatic *in vitro* culture models and is more sensitive to IFN-α than most hepatoma cell lines such as HepG2 or HepG2-NTCP. The expression of ISGs after IFN-α stimulation is partially incompetent in hepatoma cell lines. ISGs including GBP5, GBP1, WARS, and CXCL10, which have been reported to be associated with the IFN-α response in patients (118, 119), are either absent or weakly expressed in HepG2-NTCP cells treated by IFN-α (55).

## Strategies to Improve the Efficacy of IFN-Based Therapy

**Figure 1:**
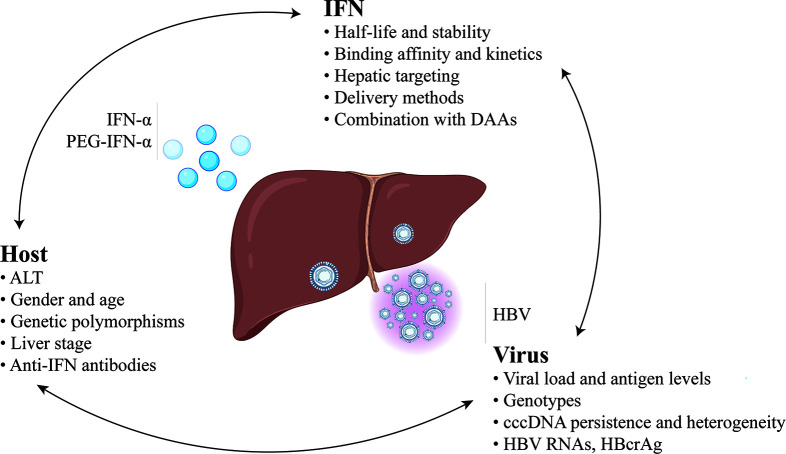
Predictors and strategies for improvement of the efficacy of IFN-based therapy for chronic hepatitis B.

Given the unique advantages of but relatively low response rates to IFN therapy, there is an urgent need to improve its efficacy (**Figure 1**).

Massive and persistent transcription of cccDNA contribute to the inhibition of both innate and adaptive immune responses of IFN. This may explain why the decrease in HBV viremia is not seen earlier than 3-4 weeks into IFN therapy and takes several months to reach its maximum (120). Thus, reduction of the viral load before IFN therapy, including HBV DNA and antigen, could be beneficial. Earlier studies showed that concomitant administration of PEG-IFN and NAs resulted in higher rates of on-treatment virologic response but had no advantage on post-therapy response compared with PEG-IFN monotherapy (57). Nonetheless, the addition of PEG-IFN to ongoing NAs therapy or switching from NAs therapy to PEG-IFN monotherapy in virally suppressed patients has shown better outcomes. A prospective study in CHB patients with HBV DNA fully suppressed by long-term NAs treatment showed that the addition of PEG-IFN for a maximum of 96 weeks led to HBsAg loss and cessation of NAs treatment in 6 of 10 patients, with no relapse for 12-18 months of follow up (121). A matched-pair study in HBeAg-positive patients who did not achieve HBeAg seroconversion after NA monotherapy showed that PEG-IFN combined with NA for another 48 weeks achieved more HBeAg seroconversion than continuing NA monotherapy (44% *versus* 6%) (122). In a randomized open-label trial, switching to a finite course of PEG-IFN significantly increased rates of HBeAg seroconversion and HBsAg loss among patients who achieved virologic suppression with NA therapy (8). Such a treatment concept can provide limited but reliable optimization in current IFN therapy.

Criteria identifying CHB patients who are suitable for IFN therapy is another critical aspect of optimization. Simple and clear guidance is needed to select specific groups of patients. At present, the natural history of CHB has been schematically divided into several phases, mainly focusing on the presence of HBeAg, HBsAg, HBV DNA levels, ALT values, and eventually the presence or absence of liver inflammation. Among them, baseline high ALT and low HBV DNA are the IFN pretreatment predictors of IFN response. Other than these classical biomarkers, novel host factors may potentially help to predict IFN treatment efficacy. For example, baseline quantitative anti-HBc titer has been shown to be a predictor of Peg-IFN efficacy in HBeAg-positive CHB patients (123). In another study, a simplified scoring model composed of miR-210, miR-22, and ALT was used to predict the virologic response of IFN therapy in CHB (124). Environmental factors and individual differences could cause a severe bias towards the accuracy of prediction. Moreover, some commonly used biomarkers, such as HBV DNA and ALT, are time-dependent and the immune states of CHB patients can hardly be reflected in a single test. For those falling into an indeterminate gray area, an explicit decision is difficult. In recent years, several studies indicate that some novel viral parameters have a statistical relationship with the response of IFN therapy, including serum HBV RNA (125, 126), hepatitis B core-related antigen (HBcrAg) (127), and spliced HBV variants (75), which are worthy of further studies in larger cohorts.

Optimization of IFN itself may, as well, be beneficial for improving therapeutic effects (128, 129). PEG-IFN-α2 is one of the most successful modified protein drugs to date. Grafting IFN-α with PEG attenuates renal filtration, and thus, decreases the rate of IFN clearance from serum. Compared with IFN-α2, PEG-IFN-α2 not only reduces the number of injections but also slightly improves the patient compliance and response rates (130). It is worth noting that poly amino acids are a group of unstructured repetitive segments of hydrophilic amino acids whose biophysical properties are similar to PEG including PAS polypeptides and elastin-like polypeptide. They have been widely considered as potential alternatives to PEG for IFN modification because of their excellent biodegradability and relatively simple preparation procedures compared with pegylation (131, 132). Apart from half-life extension, another modification aspect is the reduction of severe side-effects of IFN-α by targeting hepatocytes precisely because IFN-α acts on almost all human nucleated cells evoking a complex reaction pattern when administered systemically. Novel delivery methods that can improve targeting are of particular interest. One strategy is utilizing the metabolic characteristics of the liver by linking moieties with liver tropism to IFN-α. For instance, high-density lipoproteins (HDLs) are generated in the liver and remove cholesterol from peripheral tissues for delivery to hepatocytes. Anchoring IFN-α to ApoA-I, the main protein component of HDLs, promoted targeting to the liver, and therefore, showed increased immunostimulatory properties and lower hematological toxicity (133). In addition, orally administrable low molecular weight agent which can mimic IFN activity has been shown to suppress HBV replication and reduce cccDNA levels (134).

Increasing binding affinity between IFN-α and IFNAR has proved to be an alternative direction to improve the IFN-α biological efficacy. The key point is that a change in the affinity for IFNAR translates differently among pleiotropic activities induced by IFN-α, including antiproliferative, antiviral, and immunomodulatory activities, which means higher therapeutic effects and lower side effects compared with wild type IFN-α (135). Patten and colleagues constructed the IFN-B9X series consisting of 15 mutants by gene shuffling and point mutation (136). All 15 mutants displayed increased antiviral potency without obvious change in antiproliferative activity compared with IFN-α2. A four-residue motif (FLFY) that overlapped with the IFNAR1 binding site greatly improved the binding affinity between IFN-B9X and IFNAR1 contributed significantly to this phenotype (136). The pegylated form of IFN-B9X, which is called IFN-R7025, has exhibited ~50-fold higher anti-HCV activity compared to PEG-IFN-α2a, but only 2- to 10-fold greater antiproliferative activity *in vitro *(137). However, increasing binding affinity by gene shuffling or point mutation may generate new potential T cell epitopes, which can eventually hinder clinical transformation once it happens (138). IFN-β has the highest binding affinities for both IFNAR1 and IFNAR2. However, IFN-β is more toxic in patients, probably because of its high antiproliferative activity (139), and thus might have a low risk/benefit ratio. Among 13 human IFN-α subtypes, IFN-α2 is most widely applied in clinical investigation and treatment. However, there are studies revealing that other IFN-α subtypes with higher binding affinity towards IFNAR1 may exert better therapeutic effects in treating certain types of virus compared with IFN-α2 (140). IFN-α subtype 14, one of the naturally engineered variants, has been identified as the most potent subtype against HBV. By comparing the regions between IFN-α2 and IFN-α14 that account for the binding of IFN to IFNAR1, a variant comprised of mutations D83E, T87I, Y90F, and R121K (IFN-α2-EIFK), was constructed, which exhibited potent activity in reducing HBs and HBeAg like IFN-α14. Moreover, a concerted IFN-α and -γ response in liver, which could be efficiently elicited by IFN-α14, is associated with potent HBV suppression (141).

In addition to type I IFN, type III IFNs, consisting of four IFN-λ subtypes (IFN-λ1, IFN-λ2, IFN-λ3, and IFN-λ4), has been considered as an alternative in treatment of CHB (142). IFN-III signals through a heterodimeric receptor composed of IFN-λ receptor-1 (IFNLR1) and interleukin-10 receptor subunit beta (IL10RB) (143). IFNLR1 is expressed primarily on epithelial cells, such as hepatocytes, and on select immune cells, including pDCs and some B-lymphocytes, which may indicate better cell-type specific activity (144, 145). One of the most impactful findings about IFN-λ is the strong association of IFN-λ polymorphisms with chronic HCV clearance during the acute stage of infection and of achieving HCV cure with IFN-I-based therapy in chronic infection (146). Unfortunately, the similar association cannot be identified in HBV patients with high confidence and good reproducibility among various studies (147, 148). Although IFN-λ can activate IFN signaling pathways and lower HBV viral load, pegylated IFN-λ1 was less efficient than PEG-IFN-α2 24 weeks post-treatment because fewer patients achieved HBeAg seroconversion (149).

## Perspective

All CHB patients are at risk of progression to cirrhosis and HCC. Because of HBV cccDNA persistence and HBV DNA integration into the host genome, it has not yet been possible to eradicate HBV completely with available antiviral agents. Serum HBV DNA and HBsAg loss and sustained intrahepatic cccDNA silencing could significantly reduce the risk of the above hepatic diseases, which can be achieved in a few CHB patients receiving IFN therapy. Given that chronic HBV infection leads to immune injury and tolerance, IFN therapy, as an immune-based approach, has unique mechanistic advantages, compared with NAs, in antiviral immune modulation and may disrupt immune tolerant states. Despite a variety of direct-acting antiviral agents (DAA) targeting various steps of the HBV life cycle are underway, such as HBV entry inhibitor, viral gene expression inhibitor, capsid assembly modifiers, it seems that none of them alone will effectively cure CHB patients in the foreseeable future (150–152). A combination of IFN with these new DAAs may have synergistic effects in CHB. Thus, as discussed above, IFN therapy needs more novel and reliable biomarkers to improve clinical management, and novel combination strategies, and optimization of IFN itself, such as new IFN subtypes and delivery methods, are anticipated to substantially increase the efficacy of treatment for chronic hepatitis B.

## Author Contributions

All authors listed have made a substantial, direct, and intellectual contribution to the work and approved it for publication.

## Funding

This work was supported by National Natural Science Foundation of China (82022043, 81974304), the Shanghai Rising-Star Program (20QA1400700) and “Fuqing Scholar” Student Scientific Research Program of Shanghai Medical College, Fudan University (FQXZ202109B).

## Conflict of Interest

The authors declare that the research was conducted in the absence of any commercial or financial relationships that could be construed as a potential conflict of interest.

## Publisher’s Note

All claims expressed in this article are solely those of the authors and do not necessarily represent those of their affiliated organizations, or those of the publisher, the editors and the reviewers. Any product that may be evaluated in this article, or claim that may be made by its manufacturer, is not guaranteed or endorsed by the publisher.
